# Socioeconomic factors and self-reported health outcomes in African Americans with rheumatoid arthritis from the Southeastern United States: the contribution of childhood socioeconomic status

**DOI:** 10.1186/s12891-016-0882-5

**Published:** 2016-01-12

**Authors:** Antoine R. Baldassari, Rebecca J. Cleveland, My-Linh N. Luong, Beth L. Jonas, Consortium for the Longitudinal Evaluation of African Americans with Early Rheumatoid Arthritis, Doyt L. Conn, Larry W. Moreland, S. Louis Bridges, Leigh F. Callahan

**Affiliations:** Thurston Arthritis Research Center, University of North Carolina at Chapel Hill, CB 7280 UNC, Chapel Hill, NC 27599 USA; Department of Epidemiology, University of North Carolina at Chapel Hill, Chapel Hill, NC USA; Department of Medicine, University of North Carolina at Chapel Hill, Chapel Hill, NC USA; Emory University, Atlanta, GA USA; University of Pittsburgh, Pittsburgh, PA USA; University of Alabama at Birmingham, Birmingham, AL USA; Department of Social Medicine, University of North Carolina at Chapel Hill, Chapel Hill, NC USA

## Abstract

**Background:**

There is abundant evidence that low socioeconomic status (SES) is associated with worse health outcomes among people with Rheumatoid Arthritis (RA); however, the influence of socioeconomic disadvantage in early life has yet to be studied within that population.

**Methods:**

Data originated from the cross-sectional arm of the Consortium Evaluation of African-Americans with Rheumatoid Arthritis (CLEAR II), which recruited African-Americans with RA from six sites in the Southeastern United States. We used linear regression models to evaluate associations of parental homeownership status and educational level at participant time of birth with participant-reported fatigue (Visual Analog scale, cm), pain (Visual Analog scale, cm), disability (Health Assessment Questionnaire) and helplessness (Rheumatology Attitudes Index), independently of participant homeownership status and educational level. Models included random effects to account for intra-site correlations, and were adjusted for variables identified using backward selection, from: age, disease-duration, sex, medication use, body-mass index, smoking history.

**Results:**

Our sample included 516 CLEAR II participants with full data on demographics and covariates. 89 % of participants were women, the mean age was 54.7 years and mean disease duration was 10.8 years. In age adjusted models, parental non-homeownership was associated with greater fatigue (β = 0.75, 95 % CI = 0.36–1.14), disability (β = 0.12, 95 % CI = 0.04–0.19) and helplessness (β = 0.12, 95 % CI = 0.03–0.21), independently of participant homeownership and education; parental education had a further small influence on self-reported fatigue (β = 0.20, 95 % CI = 0.15–0.24).

**Conclusions:**

Parental homeownership, and to a small extent parental education, had modest but meaningful relationships with self-reported health among CLEAR II participants.

**Electronic supplementary material:**

The online version of this article (doi:10.1186/s12891-016-0882-5) contains supplementary material, which is available to authorized users.

## Background

There is abundant evidence that markers of low socioeconomic status (SES) are associated with worse self-reported health status [1–9] and greater disease activity [1, 3, 4, 6, 9] among people with rheumatoid arthritis (RA), who number an estimated 1.3 million in the United States. Pathways underlying these inequities are hypothesized to include and extend beyond variations by SES in health behaviors [10], occupational or environmental exposures, and in the access to, utilization and receipt of health services [11, 12].

Early-life socioeconomic disadvantage has become increasingly studied as a lasting determinant of health [13, 14]. Data in RA suggest that low SES during childhood modestly increases the later risk of disease [15–18]; illustratively, Parks and colleagues found that adverse socioeconomic conditions in early-life increase susceptibility to RA provided that SES remains low into later life-stages [17]. These findings have yet to be corroborated with data relating RA outcomes and early-life SES, as is progressively being shown in other chronic inflammatory disorders, including cardiovascular diseases [19] and type II diabetes [20].

RA studies remain predominantly focused on participants of European ancestry, and there is comparatively little research on African Americans with RA. This is especially salient in health disparities research, where the complex relations between race, gender and SES [21] compromise the generalizability of findings across racial groups in the United States. Our study aims to investigate the contribution of socioeconomic status in early life to current health outcomes in a cross-sectional sample of African Americans with RA, independently of current SES.

## Methods

### Study population

The Consortium for the Longitudinal Evaluation of African Americans with Early Rheumatoid Arthritis Registry (CLEAR) was established by the National Institute of Arthritis and Musculoskeletal and Skin Disease (NIAMS) in order to provide the research community with thorough data on traditionally under-represented African Americans. Additional information about the CLEAR consortium can be found in the literature [22] and online [23].

**Fig. 1:**
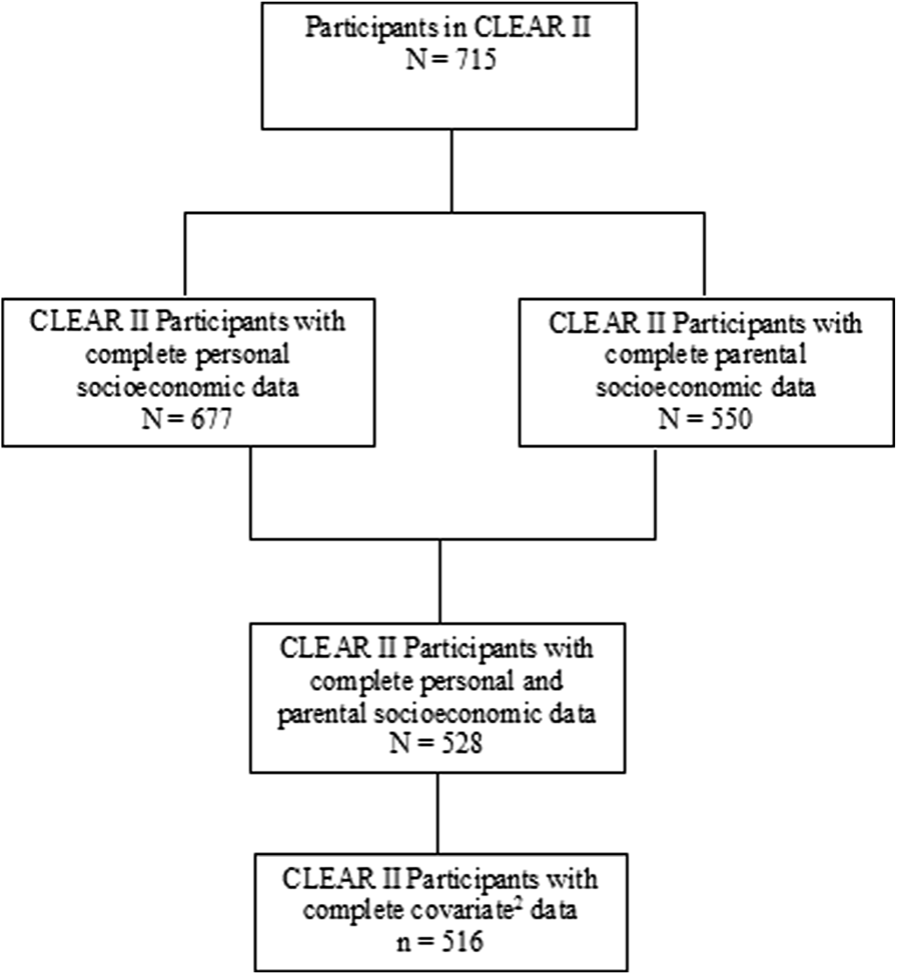
Flow chart of CLEAR^1^ II participants included in this study. ^1^ Consortium for the Evaluation of African-Americans with Rheumatoid Arthritis (RA). ^2^ Covariates include age, gender, BMI, disease duration, cigarette smoking, methotrexate use and biologic agent use

This study focuses on the cross-sectional arm of the registry, CLEAR II, which collected parental socioeconomic data. Self-identified African-Americans over the age of 18 qualified for enrollment if they provided informed consent, had RA following the American College of Rheumatology definition, and no concurrent rheumatic disease diagnostic. Recruitment took place between 2006 and 2011 at the University of Alabama at Birmingham (Birmingham, Alabama), Emory University (Atlanta, Georgia), the Medical University of South Carolina (Charleston, South Carolina), the University of North Carolina at Chapel Hill (Chapel Hill, North Carolina), and Washington University (St. Louis, Missouri). The present study includes all CLEAR II participants with complete data on sociodemographic information (*n* = 516, Fig. 1). CLEAR II was approved by the institutional review boards of each of the five participating institution listed above. All materials and methods for this study were approved by University of North Carolina at Chapel Hill biomedical institutional review board.

### Measures

#### Socioeconomic status

Current and childhood SES were assessed for participants and for their parents at the time of participant childhood, using educational and homeownership data collected in CLEAR II questionnaires.

Participant education was collected by asking “What is the highest degree or level of school you have completed?” and was dichotomized as greater than high-school (some college, but no degree; associate’s degree; bachelor’s degree; graduate school or degree; postgraduate school or degree) or high school and lower (8th grade or less; some high school; no diploma; high school graduate or equivalent;). Current homeownership, collected by asking “Do you own your own home?”, was classified as homeowner (Yes), or non-homeowners (No).

CLEAR II questionnaires collected parental education for both parents, asking participants to recall the highest grade of schooling their mother and father had completed when they were born. Data on the father was missing for one in four respondents, and we used maternal education to represent this dimension of childhood SES, when available. Where data was missing on the mother, we used father’s education (N = 10), or education of designated primary caretaker absent of data on either parent (*N* = 7). Parental education was dichotomized as high-school or greater (high school graduate or equivalent; some college, but no degree; associate’s degree; bachelor’s degree; graduate school or degree; postgraduate school or degree) or lower than high school (8th grade or less, Some high school, no diploma). The different cutpoints for participant and parental education were used to maintain comparable cell sizes of approximately 50 % in high and low education categories, and reflect the rapid rise in high-school graduation rates throughout the first half of the twentieth century [24].

Parental homeownership was collected by asking “When you were a child, did your parents, or the persons who raised you, own or were they buying their home, paying rent, or did they have some other living arrangement, such as living with relatives?” and it was classified, as for participant homeownership, as homeowner (Own or buying), or non-homeowner (Paying rent, Other living arrangement).

#### Self-reported health status

Self-reported health outcomes included in this study were the Health Assessment Questionnaire (HAQ), the helplessness subscale of the Rheumatology Attitudes Index (RAI), and Visual Analog Scale ratings for pain and fatigue (VAS).

The HAQ is a validated disability index widely-used in the monitoring of rheumatic diseases [25]. It consists of 20 items assessing a patient’s functional ability in eight domains of daily living: dressing and grooming (2 items), arising (2 items), eating (3 items), walking (2 items), hygiene (3 items), reach (2 items), grip (3 items), and usual activities (3 items). Questions are answered on a four-level scale (0: without any difficulty, 3: unable to do), and the highest ratings for each domain are averaged into a final score out of three, where higher scores indicate greater disability.

The RAI helplessness subscale has participants rate the following five statements on a 5-point integer scale (1: strongly disagree, 5: strongly agree):”My condition is controlling my life”, “I would feel helpless if I couldn’t rely on other people for help with my condition”, “No matter what I do, or how hard I try, I just can’t seem to get relief from my pain”, “I am not coping effectively with my condition”, and “It seems as though fate and other factors beyond my control affect my condition”. The five items are averaged into a final score, where higher values indicate greater levels of helplessness. The RAI is a validated indicator of perceived control over rheumatic conditions and is acceptably reliable for research and population screening purposes [26, 27].

Pain and fatigue VAS scores are produced by having participants rate their symptoms, respectively of pain or fatigue, along a continuous 10 cm line, with 0 cm corresponding to no pain/fatigue, and 10 cm meaning pain/fatigue as bad as could be. VAS measures of pain and fatigue have good test-retest reliability and internal validity [28, 29].

#### Covariates

The following variables were considered as control variables: age (continuous years); sex, body mass index (BMI, kg/m^2^); disease duration (continuous years); smoking status (never, former or current); current methotrexate/leflunomide use (yes/no) and current biologic agent use (yes/no).

#### Statistical models

We used linear regression models to examine the relationships between SES and each of the four outcome measures and included random effects to account for possible intra-site correlations. Our first model concurrently included participant education with parental education. The second model included participant homeownership and parental homeownership. The final model included all four socioeconomic factors. Backward elimination of covariates in multivariable models was carried out and change in estimate and likelihood ratio tests (LRT) were used to select covariates for the final model.

We report parameter estimates (β) and their 95 % confidence intervals (CI) comparing low-SES to high-SES (reference) for each outcome variable, as well as p-values from the LRT comparing the first two models to that including all four SES measures. Tests of statistical significance were two-sided and are considered significant at the *p* = 0.05 level. All statistical analyses were completed using the statistical software package SAS 9.4 (SAS Institute Inc. Cary, NC).

## Results

Models adding covariates beyond age were not found to meaningfully improve on age-adjusted models (*p* < 0.05). Consequently, we present our results adjusted for age alone. The tables detailing the progressive adjustments for covariates and their effect on the models may be found in the provided supplementary material (Additional file 1: Tables S1 and S2).

### Participants

Sample characteristics are shown in Table 1. Most participants were women (89 %), the mean age was 55 (SD = 11) and the mean disease duration was 11 years (SD = 10). On average, participants rated their pain (6.0 cm, SD = 2.8) and fatigue (6.3 cm, SD = 3.0) as moderately severe, and their mean scores of 1.3 on (SD = 0.7) the HAQ and 2.8 (SD = 1.0) on the RAI indicated moderate-to-severe disability and helplessness [25, 26]. Two-thirds of participants used methotrexate or leflunomide, and about a quarter used biologic agents.

**Table 1:** Characteristics of CLEAR II participants included in the study (*N* = 516)

	Mean (SD) or Percent
	Participants (N)^a^
*Demographics and lifestyle*	
Age (years)	54.7 (11.2)
Gender (% Female)	89.5
Currently smoking (%)	23.3
Disease duration (years)	10.8 (9.5)
Total smoking (pack years)	9.1 (16.2)
*Current medication use (%)*	
Methotrexate or Leflunomide	63.6
Biological agents	24.4
*Socioeconomic status (%)*	
Less than or = HS	47.5
Homeownership	56.0
Parental education < HS	55.2
Parental homeownership	43.2
*Self-reported health*	
Fatigue (VAS cm)	6.0 (3.0)
Pain (VAS cm)	6.3 (2.8)
Disability (HAQ)	1.3 (0.7)
Helplessness (RAI)	2.8 (1.0)

*CLEAR* Consortium for the Longitudinal Evaluation of African-Americans with Rheumatoid Arthritis, *SD* Standard deviation

^a^Dataset restricted to the 516 participants with complete data on SES and covariates: age, sex, body mass index, disease duration, smoking status, current methotrexate/leflunomide use, and current biologic agent use

Eighty-four percent of respondents graduated from high school and 53 % received further schooling, while fewer than half (45 %) were raised by a high-school graduate; consequently, roughly equal numbers of participants were in the low and high SES categories for both participant and parental education. Parental homeownership during participant childhood was at 57 %, and 44 % of all participants were themselves homeowners.

### Regression results

#### Model 1: Parental and participant education

Results from all three linear models may be found in Table 2. Participants with only a high-school degree or less reported meaningfully worse health across all self-reported instruments compared with those in education categories beyond high school, with the differences at 0.29 on the HAQ index (95 % CI: 0.18, 0.40), 0.99 cm (95 % CI: 0.49, 1.48) and 0.45 cm (95 % CI: 0.09, 0.81) on respectively pain and fatigue VAS, and 0.23 on the RAI (95 % 0.13, 0.33). The relationship between parental education and self-reported health was comparatively weak, with individuals raised by parents without a high school degree rating their fatigue 0.21 cm higher (95 % CI: 0.16, 0.25) but otherwise reporting similar disability, pain and helplessness as their high childhood-SES counterparts.

**Table 2:** Associations of participant and parental low-Socioeconomic status markers with self-reported health outcomes in CLEAR II^a^

	Current education^b^	Parental education^c^	Current homeownership^d^	Parent homeownership^d^	LRT p vs. full
*Model 1*					
Fatigue (VAS cm)	**0.45 (0.09,0.81)**	**0.21 (0.16,0.25)**			0.06
Disability (HAQ)	**0.29 (0.18,0.40)**	**−0.07 (−0.12,-0.02)**			0.13
Pain (VAS cm)	**0.99 (0.49,1.48)**	0.06 (−0.15,0.27)			0.20
Helplessness (RAI)	**0.23 (0.13,0.33)**	0.09 (−0.10,0.27)			**0.04**
*Model 2*					
Fatigue (VAS cm)			0.29 (−0.09,0.66)	**0.82 (0.43,1.20)**	0.85
Disability (HAQ)			**0.15 (0.10,0.20)**	**0.16 (0.09,0.24)**	**0.01**
Pain (VAS cm)			**0.58 (0.27,0.89)**	0.58 (−0.04,1.19)	**0.03**
Helplessness (RAI)			**0.27 (0.16,0.38)**	**0.16 (0.08,0.25)**	0.22
*Model 3*					
Fatigue (VAS cm)	0.22 (−0.13,0.58)	**0.20 (0.15,0.24)**	0.27 (−0.08,0.62)	**0.75 (0.36,1.14)**	
Disability (HAQ)	**0.24 (0.14,0.35)**	**−0.06 (−0.11,-0.01)**	**0.11 (0.08,0.14)**	**0.12 (0.04,0.19)**	
Pain (VAS cm)	**0.82 (0.33,1.30)**	0.08 (−0.14,0.29)	**0.46 (0.18,0.75)**	0.40 (−0.24,1.04)	
Helplessness (RAI)	**0.16 (0.06,0.27)**	0.10 (−0.08,0.29)	**0.25 (0.15,0.36)**	**0.12 (0.03,0.21)**	

*CLEAR* Consortium for the Longitudinal Evaluation of African-Americans with Rheumatoid Arthritis, *HAQ* Health Assessment Questionnaire, *LRT* Likelihood Ratio Test, *VAS* Visual analog scale (in centimeters), *RAI* Rheumatology Attitudes Index

Bolded results are statistically significant at the α = 0.05 level

^a^Adjusted for age; dataset restricted to the 516 participants with complete data on SES and covariates: age, sex, body mass index, disease duration, smoking status, current methotrexate/leflunomide use, and current biologic agent use

^b^Participant low education: ≤HS, compared to high education (>HS)

^c^Parental low education: <HS, compared to high education (≥HS)

^d^Non-homeownership, compared to homeownership

#### Model 2: Parental and participant homeownership

Parental and participant non-homeownership were both associated with modest increases in disability (respectively HAQ β = 0.16 [95 % CI: 0.09, 0.24] and β = 0.15 [95 % CI: 0.10, 0.20]) and helplessness (β = 0.27 [95 % CI: 0.16, 0.38] and β = 0.16 [95 % CI: 0.08, 0.25]). Further, homeowners rated their pain 0.58 cm lower than non-homeowners (β = 0.58 [95 % CI: 0.27, 0.89]), and participants raised by non-homeowners rated their fatigue 0.82 cm (95 % CI: 0.43, 1.20) higher than individuals reared in owner-occupied homes.

#### Model 3: Full model

In models simultaneously including parental and participant homeownership along with parental and participant education, respondents in the low education category still had measurably greater disability (β = 0.24 [95 % CI: 0.14, 0.35]), pain (β = 0.82 [95 % CI: 0.33, 1.30]) and helplessness (β = 0.16 [95 % CI: 0.06, 0.27]) but no longer significantly greater fatigue than those with education past high school. In contrast, there remained no discernable associations between parental education and health outcomes. As in the earlier models, non-homeowners scored 0.11 higher on the HAQ (95 % CI: 0.08, 0.14), rated their pain 0.46 cm higher (β = 0.18, 0.75) and scored 0.25 higher on the RAI (β = 0.15, 0.36) than homeowners. Individuals whose parents did not own a home during their childhood experienced greater fatigue (β = 0.75 [95 % CI: 0.36, 1.14]), disability (β = 0.12 [95 % CI: 0.04, 0.19]) and helplessness (β = 0.12 [95 % CI: 0.03, 0.21]) than those raised in owner-occupied homes.

## Discussion

Our results suggest there are modest variations in self-reported health by parental SES during childhood, within this cohort of African Americans with RA. This was most notable with regards to fatigue, where the adjusted effect of parental homeownership approached the minimally important clinical difference, estimated to range between 0.82 and 1.1 cm [30, 31]. Smaller differences in disability and helplessness were also observed by parental homeownership status, independently of participant SES.

While past research suggests a link between early-life SES and the onset of RA, this current study is the first to investigate differences in health outcomes by early SES among individuals with RA. Our focus on African-Americans further distinguishes our analyses from prior work on disparities in RA; to some extent, our results may be specific to this demographic group, considering the complex relationships between SES, race and health in the United States [21].

Our finding that parental homeownership was associated with health outcomes ahead of parental education may offer clues as to the proximal pathways linking childhood SES to health among CLEAR participants. The range of physical and psychological mechanisms that could account for our result remains vast, however. For instance, parental homeownership is a marker of a family’s material resources, and hence of the access to and quality of health care available in early-life – a direct determinant of childhood health likely to reverberate into later life stages. Key characteristics of the childhood physical environment, ranging from food security, crowding and salubrity, to exposure to pollutants vary according to markers of wealth [30], and could further account for the health differences we observed. In addition, socioeconomic insecurity during childhood is psychologically disruptive and may notably promote learned helplessness [32] a suspected correlate of disability, pain and fatigue scores among individuals with inflammatory arthritis [33]. Mounting evidence further suggests that chronic stressors such as sustained economic disadvantage during childhood trigger biological adaptations detrimental to long-term health; of particular relevance in inflammatory illnesses such as RA, there is a forming consensus that individuals raised in disadvantaged environments lastingly exhibit altered immune functioning and exaggerated inflammation [34].

Study limitations included the crudeness of our SES measures, which were dichotomized to allow for acceptable cell sizes and therefore could not discern gradient patterns of health inequalities. In addition, participant recall of parental homeownership and education may be influenced by personality traits correlated with worse health perceptions [35]. Further, differences in self-reported health by SES could indicate differences in reporting behavior rather than in underlying health status, although there is data to suggest this may bias estimates of childhood SES and health towards the null [36]. Finally, whereas education changes little after early adulthood, homeownership status may be affected by poor health, for instance due to functional decline restricting ability to work, allowing for a reverse causal relationship to occur.

## Conclusions

Our findings constitute evidence that socioeconomic differences in the health of individuals with RA take their roots early in the lifecourse. Although the effect size was modest, we encourage physicians to be cognizant of RA patients’ socioeconomic background over multiple life stages in the monitoring of their health.

## Additional file

Additional file 1:
**Table S1:** Parameter estimates and 95 % confidence intervals for the associations of participant and parental low education level with self-reported health outcomes in the CLEAR II registry, with progressive adjustment for covariates. **Table S2:** Parameter estimates and 95 % confidence intervals for the associations of participant and parental non-homeownership with self-reported health outcomes in the CLEAR II registry, with progressive adjustment for covariates. (DOCX 17 kb)

## Abbreviations

CLEAR: Consortium for the Longitudinal Evaluation of African Americans with Rheumatoid Arthritis
HAQ: Health Assessment Questionnaire
LRT: Likelihood Ratio Test
RA: Rheumatoid Arthritis
RAI: Rheumatology Attitudes Index
SES: Socioeconomic Status
VAS: Visual Analog Scale
