# Supplementary material for: Socioeconomic factors and self-reported health outcomes in African Americans with rheumatoid arthritis from the Southeastern United States: the contribution of childhood socioeconomic status
Source: BMC Musculoskelet Disord. 2016 Jan 12;17:10. doi: 10.1186/s12891-016-0882-5 (PMC4709946; doi:10.1186/s12891-016-0882-5)
Supplement: Additional file 1: — Table S1: Parameter estimates and 95 % confidence intervals for the associations of participant and parental low education level with self-reported health outcomes in the CLEAR II registry, with progressive adjustment for covariates. Table S2: Parameter estimates and 95 % confidence intervals for the associations of participant and parental non-homeownership with self-reported health outcomes in the CLEAR II registry, with progressive adjustment for covariates. (DOCX 17 kb) [file 12891_2016_882_MOESM1_ESM.docx]

**Table S1.** Parameter estimates and 95% confidence intervals for the associations of participant and parental low education level with self-reported health outcomes in the CLEAR II registry, with progressive adjustment for covariates (N=516**^1^**).

|  |  | Participant | Parent |  |
| --- | --- | --- | --- | --- |
|  |  | Education^2^ | Education^3^ | LRT p-value^4^ |
| Fatigue VAS, cm | Step 1 | **0.41 (0.02,0.80)** | **0.20 (0.13,0.27)** |  |
|  | Step 2 | **0.44 (0.08,0.79)** | **0.21 (0.16,0.25)** | 0.99 |
|  | Step 3 | **0.45 (0.09,0.81)** | **0.21 (0.16,0.25)** | 0.98 |
|  |  |  |  |  |
| Disability (HAQ) | Step 1 | **0.29 (0.18,0.40)** | **-0.07 (-0.12,-0.03)** |  |
|  | Step 2 | **0.29 (0.18,0.40)** | **-0.07 (-0.12,-0.02)** | 0.62 |
|  | Step 3 | **0.29 (0.18,0.40)** | **-0.07 (-0.12,-0.02)** | 0.61 |
|  |  |  |  |  |
| Pain VAS cm | Step 1 | **0.90 (0.33,1.47)** | 0.09 (-0.10,0.28) |  |
|  | Step 2 | **0.98 (0.48,1.48)** | 0.06 (-0.15,0.27) | 0.82 |
|  | Step 3 | **0.99 (0.49,1.48)** | 0.06 (-0.15,0.27) | 0.85 |
|  |  |  |  |  |
| Helplessness (RAI) | Step 1 | **0.21 (0.10,0.32)** | 0.09 (-0.10,0.28) |  |
|  | Step 2 | **0.23 (0.12,0.33)** | 0.09 (-0.10,0.28) | 0.85 |
|  | Step 3 | **0.23 (0.13,0.33)** | 0.09 (-0.10,0.27) | 0.78 |

VAS: Visual analog scale (in centimeters), HAQ: Health Assessment Questionnaire, LRT: likelihood ratio test

Bolded results indicate statistical significance at the α = 0.05 level

## ^1^ Dataset restricted to the 516 participants with complete data on SES and all covariates: age, sex, body mass index, disease duration, smoking status, current methotrexate/leflunomide use, and current biologic agent use

^2^ Participant low education: ≤HS, compared to high education (>HS)

^3^ Parental low education: <HS, compared to high education (≥HS)

^4^ Compared to step 1

Step 1: Fully adjusted model (age, sex, BMI, disease duration, pack-years of smoking, Methotrexate or Leflunomide use and biologic agent use)

Step 2: Reduced model (age and disease duration)

Step 3: Further reduced model (age only)

## Table S2. Parameter estimates and 95% confidence intervals for the associations of participant and parental non-homeownership with self-reported health outcomes in the CLEAR II registry, with progressive adjustment for covariates (N=516^1^).

|  |  | Participant | Parental |  |
| --- | --- | --- | --- | --- |
|  |  | Homeownership^2^ | Homeownership^2^ | LRT p-value^3^ |
| Fatigue (VAS cm) | Step 1 | 0.27 (-0.17,0.71) | **0.85 (0.46,1.24)** | ref |
|  | Step 2 | 0.29 (-0.09,0.67) | **0.84 (0.45,1.22)** | 0.97 |
|  | Step 3 | 0.29 (-0.09,0.66) | **0.82 (0.43,1.20)** | 0.95 |
|  |  |  |  |  |
| Disability (HAQ) | Step 1 | **0.14 (0.08,0.21)** | **0.16 (0.09,0.24)** | ref |
|  | Step 2 | **0.15 (0.10,0.20)** | **0.16 (0.09,0.23)** | 0.61 |
|  | Step 3 | **0.15 (0.10,0.20)** | **0.16 (0.09,0.24)** | 0.65 |
|  |  |  |  |  |
| Pain (VAS cm) | Step 1 | **0.57 (0.24,0.91)** | **0.63 (0.06,1.20)** | ref |
|  | Step 2 | **0.59 (0.28,0.90)** | 0.59 (-0.03,1.20) | 0.56 |
|  | Step 3 | **0.58 (0.27,0.89)** | 0.58 (-0.04,1.19) | 0.57 |
|  |  |  |  |  |
| Helplessness (RAI) | Step 1 | **0.27 (0.14,0.39)** | **0.17 (0.09,0.25)** | ref |
|  | Step 2 | **0.27 (0.17,0.38)** | **0.17 (0.08,0.25)** | 0.75 |
|  | Step 3 | **0.27 (0.16,0.38)** | **0.16 (0.08,0.25)** | 0.61 |

VAS: Visual analog scale (in centimeters), HAQ: Health Assessment Questionnaire, LRT: likelihood ratio test

Bolded results indicate statistical significance at the α = 0.05 level

## ^1^ Dataset restricted to the 516 participants with complete data on SES and all covariates: age, sex, body mass index, disease duration, smoking status, current methotrexate/leflunomide use, and current biologic agent use

## ^2^ Non-homeownership compared to homeownership

^3^ Compared to Step 1

Step 1: Fully adjusted model (age, sex, BMI, disease duration, pack-years of smoking, Methotrexate or Leflunomide use and biologic agent use)

Step 2: Reduced model (age and disease duration)

Step 3: Further reduced model (age only)
